# An Evaluation of Posture Recognition Based on Intelligent Rapid Entire Body Assessment System for Determining Musculoskeletal Disorders

**DOI:** 10.3390/s20164414

**Published:** 2020-08-07

**Authors:** Ze Li, Ruiqiu Zhang, Ching-Hung Lee, Yu-Chi Lee

**Affiliations:** 1 School of Design, South China University of Technology, Guangzhou 510641, China; 201821043324@mail.scut.edu.cn (Z.L.); rqzhang@scut.edu.cn (R.Z.); 2 School of Public Policy and Administration, Xi’an Jiaotong University, Xi’an 710000, China; leechinghung@xjtu.edu.cn

**Keywords:** ergonomics, rapid entire body assessment (REBA), convolutional pose machines, posture analysis, musculoskeletal disorders (MSDs)

## Abstract

Determining the potential risks of musculoskeletal disorders through working postures in a workplace is expensive and time-consuming. A novel intelligent rapid entire body assessment (REBA) system based on convolutional pose machines (CPM), entitled the Quick Capture system, was applied to determine the risk levels. The aim of the study was to validate the feasibility and reliability of the CPM-based REBA system through a simulation experiment. The reliability was calculated from the differences of motion angles between the CPM-based REBA and a motion capture system. Results show the data collected by the Quick Capture system were consistent with those of the motion capture system; the average of root mean squared error (RMSE) was 4.77 and the average of Spearman’s rho (ρ) correlation coefficient in the different 12 postures was 0.915. For feasibility evaluation, the linear weighted Cohen’s kappa between the REBA score obtained by the Quick Capture system and those from the three experts were used. The result shows good agreement, with an average proportion agreement index (P_0_) of 0.952 and kappa of 0.738. The Quick Capture system does not only accurately analyze working posture, but also accurately determines risk level of musculoskeletal disorders. This study suggested that the Quick Capture system could be applied for a rapid and real-time on-site assessment.

## 1. Introduction

With the development of science and technology, occupational diseases are gradually controlled; however, musculoskeletal disorders (MSDs) are gaining more attention. Musculoskeletal disorders are a primary occupational disease related to working posture, which not only affects the health of workers but also causes enormous economic losses to countries [1,2]. In addition to chemical, physical, and biological occupational hazards, there are also many other harmful factors or loads in a workplace, such as manual lifting, static work, unreasonable working posture, and labor organization. The primary morbidity factor of MSDs is poor working posture. For example, the leading cause of carpal tunnel syndrome in automobile assembly workers is the long-term bending of hands [3]. The trunk diseases in construction workers are mainly caused by long-term bending or standing and uncomfortable lifting posture [4]. Improper working postures are directly related to the risk of MSDs, work efficiency, and security accidents.

Therefore, prevention and rapid assessment of the hazards caused by working posture and adoption of scientific corrective measures have become a vital issue, which are important for reducing the occurrence of MSDs. Sutari et al. [5] found that workers in the ‘Batik cap’ industry experienced musculoskeletal disorders of the legs, arms, and wrists; thus, they adjusted the height of the workbench to achieve a better working posture. Bazazan et al. [6] investigated for 12 months the effects of posture correction-based intervention on musculoskeletal symptoms and fatigue of control room operators, whose shoulders, upper backs, necks, and lower backs are prone to illnesses. The intervention was found to improve muscle dysfunction in workers.

### 1.1. Traditional Assessment Methods

To determine MSDs in humans, self-assessment, direct measurement, and observational assessment are commonly used methods to actively assess workers’ working posture [7]. The self-assessment method gathers unfavorable factors in the workplace through analysis of working diaries, interviews, and questionnaires [8]. Bernardes et al. [9] used the Nordic Musculoskeletal Questionnaire (NMQ) in studying 17–59-year-old workers in Botucatu, Brazil. They found from self-reports that musculoskeletal symptoms of upper limbs and spine were related to work-related accidents. The disadvantage of the self-assessment method is that it is subjective. The direct measurement method collects musculoskeletal data and motion angles from workers through sensors, which are attached to their bodies while working [10,11]. These sensors mainly consist of motion capture systems, electroencephalographs, and motion sensors [12]. However, direct measurement methods are usually expensive to set up and time-consuming [13]. They also affect physiological and psychological performances of the participants [14]. The observational assessment is more widely used in the industry. It does not only allow workers to be observed directly and objectively during work without interference but also provides precise and accurate results for MSDs risk assessment.

To carry out observational assessment, researchers used the Ovako working posture analysis system (OWAS) [15], the rapid entire body assessment (REBA) [16], the rapid upper limb assessment (RULA) [17], and other methods that assess the risk level of work posture [18]. The REBA method is one of the most popular methods for rapid assessment of static tasks. Yoon et al. [19] used the REBA method to evaluate the workload of three types of automobile assembly lines, namely, chassis, accessories, and finishing, and formulated a work rotation plan. Janowitz et al. [20] developed and validated a modified REBA scoring algorithm for an ergonomic assessment of a hospital worker and reported an inter-rater reliability kappa of 0.54 for upper extremity and 0.66 for lower extremity. Ansari et al. [21] and Kee et al. [22] compared the difference between the REBA and RULA methods for evaluating the risk of workers’ working posture in small handicraft factories in India and Indonesia, respectively. The results of the two abovementioned studies indicated that REBA and RULA were identical. The REBA method considered all the body parts, except the foot and ankle. Hignett and McAtamney [16] evaluated the precision of REBA and found that 62–85% of expert users scored similarly for all the selected postures, except for the upper arm [20]. Schwartz et al. [23] studied eight janitorial tasks and found that the average REBA scores for all tasks were in high-risk categories. Eight observers used the REBA method to evaluate their tasks and verified the reliability of REBA in practice (ICC = 0.925). The observers were highly confident in using the REBA method after standardized training [23]. Thus, the REBA method has high accuracy, can thoroughly evaluate the risk of factory workers’ working posture, and can perform automatic evaluation through algorithms.

During the assessment process, angle data collection from each body part is a problem. Savino et al. [24] applied a video observation method to classify specific postures in a manual assembly process. This method used a substantial amount of workforce and material resources. The angle data were also affected by the visual fatigue of observers [25]. Most researchers use close-fitting data labels and specialize equipment to collect data. For example, Nath et al. [4] focused on the trunk and shoulder parts, which have the highest incidence of MSDs among construction workers. They attached a smartphone as the data acquisition equipment to the workers’ arms and waists. The smartphone sensors were used to unobtrusively monitor workers’ postures and autonomously identify potential work-related ergonomic risks. Vignais et al. [26] established a biomechanical model using inertial sensors placed at different positions on the upper body and performed a computerized RULA ergonomic evaluation. Although these collection methods have high accuracy, they also have some limitations, such as difficulty in using in practical environments and the impact of the attached sensors on the regular work of workers.

### 1.2. The State of the Art

With the advancement in machine learning, image recognition, deep learning, and computer vision, some researchers have made significant progress in posture recognition by improving the algorithms. The classical method for human joint posture assessment is image structure model [27,28,29,30], in which the spatial correlation between various body parts is represented as a tree structure graphics model. This model has been applied to restructure all extremities visibly from images obtained using a depth camera [31]. Manghisi et al. [32] used the skeletal data identified by a Microsoft Kinect V2 depth camera to recognize the working posture and entirely developed a series of working posture risk assessment systems based on RULA evaluation rules (K2-RULA). The software can detect working posture in real-time and do offline analysis. Manghisi et al. [32] conducted two experiments to verify the effect of the K2-RULA system. The accuracy of the K2-RULA posture recognition was verified by comparing it with motion capture systems. The feasibility of K2-RULA for posture assessment was examined by comparing the results of K2-RULA and ergonomics experts. Zhao et al. [33] used Kinect V2 and convolution neural networks to identify the limb angles of the human body and calculate the standard posture risk assessment score to rapidly and accurately obtain the risk assessment results of various working postures. However, this method uses a Kinect depth camera, which is more complicated to install and use than a smartphone. The algorithm in the supporting software of the depth camera requires high computing resources to implement, which results in lower resolution. A poor picture quality may affect the accuracy of joint point recognition and evaluation results.

Recently, there has been a significant interest in using a convolution network structure for the assessment of human joints postures [34,35]. The DeepPost model, proposed by Toshev and Szegedy [36], uses a deep convolution neural network for global reasoning. The model also applies standard convolution structure to directly regress Cartesian coordinates and treats posture assessment as a regression problem to solve. Oberweger et al. [37] investigated the effect of a convolutional neural network with multi-scale input on gesture recognition. By using multi-dimensional features of images, the recognition accuracy was significantly improved. Wei et al. [38] developed a sequential architecture consisting of a convolution network for posture recognition and integrated convolutional pose machines (CPM) for joint posture estimation. Abobakr et al. [39] proposed a deep convolutional neural network to predict body joint points from a single image and used angle data for MSDs risk assessment using the RULA method. From the experimental comparison between the risk level score from experts and the abovementioned neural network, the prediction accuracy obtained was 89%, the kappa index was 0.71, and the average mean absolute error (MAE) was 3.19° ± 1.57°. The application of convolution neural networks in combination with CPM is a means to identify working posture in real-time.

### 1.3. Summary of Previous Studies and Main Contributions of This Study

Previous research indicated that image recognition has unique advantages in MSDs risk assessment. However, there are three issues that need to be addressed. Firstly, the systems developed in the current research are mostly based on Kinect, but the hardware size of the Kinect is still large. In setting up Kinect, supporting data cables, power cables, and computers are required, which make its operation impractical for industrial field evaluation. Secondly, most evaluation systems are based on the REBA or RULA rules, which are manual-setting default parameters. Thus, numerous parameters, such as force, load, and shoulder abduction, are difficult to be adjusted automatically. This is the reason why current systems lead to lower scoring accuracy. Thirdly, at present, most systems related to MSDs adopt a random forest algorithm, which lacks high precision and portability compared with the CPM method. The CPM method has been successfully integrated into monitoring detectors and has proven to be an effective approach in monitoring detectors [40]. However, only few studies have been focused on CPM applications in ergonomic evaluation.

To address these research issues, the authors of this study developed a novel smartphone-based “Quick Capture” system in the previous research. It is a CPM-based REBA system for MSDs risk assessment, which integrates a rule-based human risk calculating (HRC) formula and convolutional pose machines (CPM). Thus, the main contributions of this study are as follows: (1) the automatic parameters’ judgment and evaluation were supplemented and modified to improve score accuracy of the system; (2) the reliability of CPM algorithm in the human factor evaluation system was verified; (3) the feasibility of the REBA rule in the automatic evaluation system was confirmed.

## 2. Methods

### 2.1. Quick Capture System: A CPM-Based REBA System

**Figure 1:**
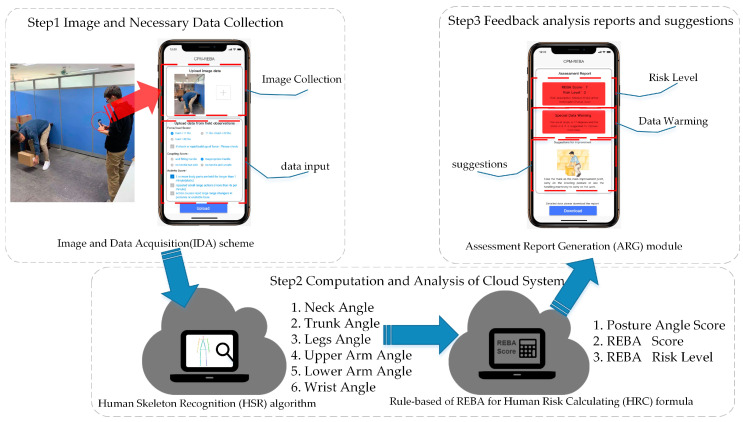
The system architecture of the Quick Capture system.

A novel smartphone-based and CPM-based REBA system, titled the “Quick Capture” system for MSDs risk assessment, was applied in the study by using C++, VS2015, php, ngrok, OpenPose Open Source File, and Windows Presentation Foundation libraries. As shown in Figure 1, the system consists of four parts: (1) an image and data acquisition (IDA) scheme; (2) a human skeleton recognition (HSR) algorithm based on CPM; (3) REBA with a rule-based human risk calculating (HRC) formula; (4) an assessment report generation (ARG) module. The core of the HSR algorithm is based on CPM, which uses a sequential convolution architecture to express the spatial and texture information of a human pose in a two-dimensional image. The network is divided into multiple stages: the initial stage uses the original picture as input, while the later stage uses the feature map of the previous stage as input. The primary purpose is to fuse spatial and texture information and center constraints. This also allows the CPM to have both image and spatial feature learning capabilities and implicit spatial modeling capabilities, which effectively solves the skeleton prediction problem under the occlusion of body parts. The authors have uploaded the CPM code to GitHub (see Appendix B). The HRC formulas and data processing are discussed in detail in the subsequent chapters.

The evaluation procedure is divided into three steps. The first step is accomplished on the IDA scheme to complete the acquisition of image data through real-time photography or by uploading a photo. Subsequently, the necessary data are entered, such as task description and basic information of the worker, among others. The image uploaded from the previous step is used as input data for the HSR algorithm on human skeleton recognition to obtain two-dimensional coordinate data for human joint points. The limb angles of the image are then calculated. Then, the angle data and task description from the first step are entered into the HRC formula to establish the human posture skeleton model and calculate the postural angle score, REBA score, and risk level of MSDs. The HSR algorithm and HRC formula are calculated using cloud computing technology. The next step is to download the MSDs assessment report, including a human posture skeleton map, REBA score, risk level result, and suggestion generated by the previous back to the smartphone. Users could quickly check the result and complete the overall assessment process.

**Figure A1:**
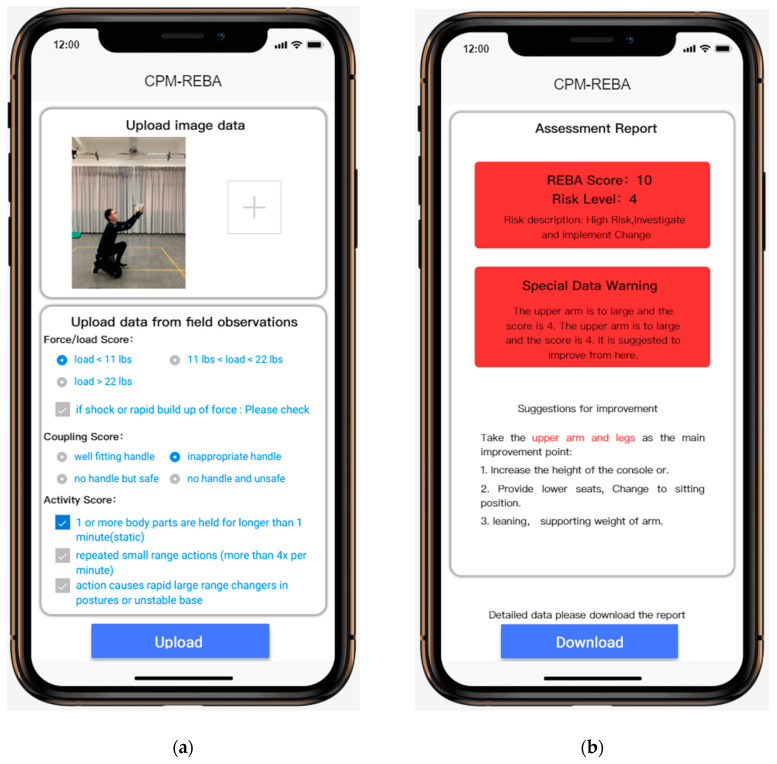
The main interface of the Quick Capture system. Data collection and upload (**a**), and REBA online assessment report (**b**).

**Figure A2:**
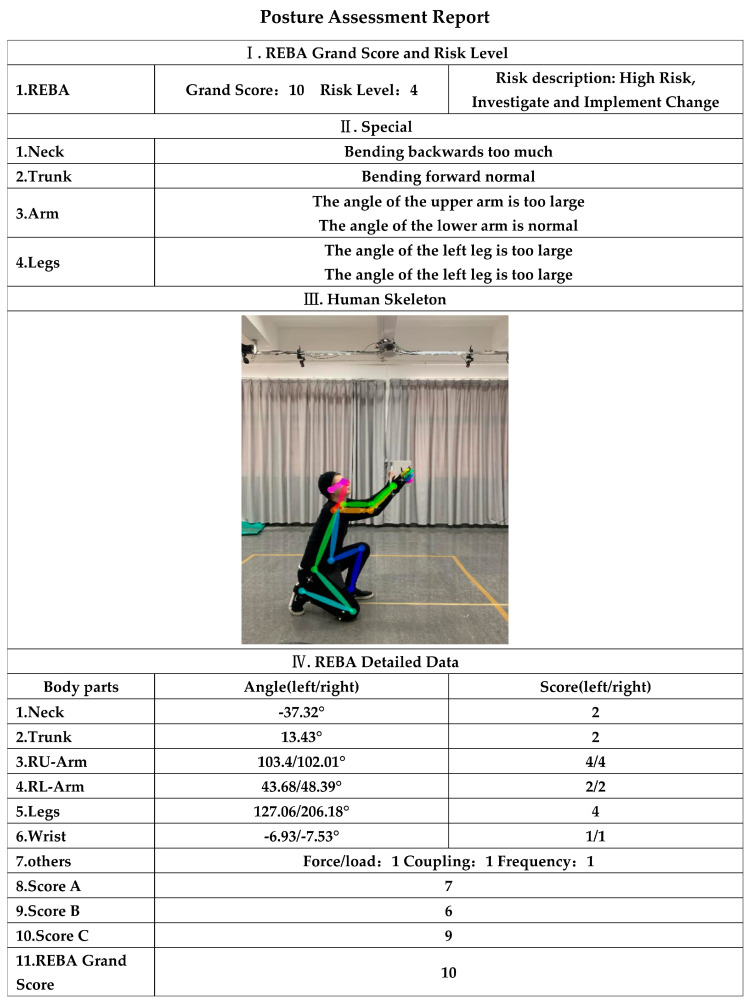
The detailed report of the Quick Capture system.

The CPM-based REBA system is easy to use. The system is packed as an application, which allows it to be installed on smartphones. The photo of the targets taken by a smartphone camera can be uploaded by one click (see Figure A1a in Appendix A). The risk assessment results for working posture evaluation are automatically sent back to the smartphone. The whole analysis can take approximately 10 s, depending on network condition. The experts can view the online report (see Figure A1b in Appendix A). The interface background is green when the risk level is less than 3 and red when it is greater than or equal to 3. They can also explore the detailed report for each body part offline (see Figure A2 in Appendix A).

#### 2.1.1. REBA with Rule-Based Human Risk Calculating (HRC) Formula

The REBA method analyzes a series of influencing factors in the process, such as body posture, force/load, coupling tools, and frequency of activities among others and obtains corresponding scores of the various influencing factors from the score sheet. The total score of REBA, which ranges from 1 to 15, is obtained to determine the risk of MSDs [16]. The REBA score is divided into five risk levels. The higher the risk level, the higher the hazard related to working posture. The rules of the REBA method are as follows. Firstly, the human body is classified into two segments (segments A: neck, trunk, and legs; segments B: upper arm, lower arm, and wrist). Tables A and B of the REBA method are used to gather the posture score of the two segments. Then, the two scores are modified according to the task description, such as fore/load and coupling condition as score A and score B, respectively. Subsequently, score C is obtained from Table C using scores A and B. Finally, score C and activity score (demand of the task) are added to obtain the final REBA score. All the REBA evaluation procedures are described in the work of Hignett and McAtamney [16]. The hazard level of working posture is assessed based on the relationship between total score and risk level (Table 1).

#### 2.1.2. Data Retrieval

**Figure 2:**
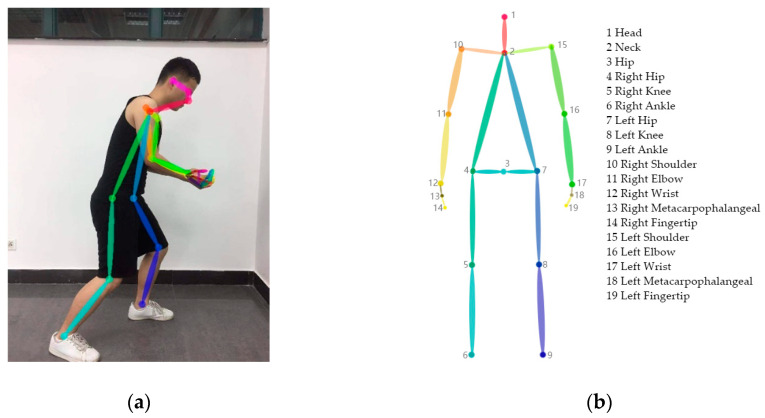
Human skeleton model provided by convolutional pose machines (CPM) (**a**), and the human skeleton model and joint point number required for evaluation (**b**).

The human skeleton structure can describe the body posture during working. Nineteen joint points related to the body posture were extracted from the position data of the joint points in the two-dimensional image acquired by the CPM of the human posture recognizer to form the human skeleton structure, as shown in Figure 2.

Although the HSR can obtain two-dimensional position coordinates for postural joint points from pictures or videos, the position of joint points cannot be directly entered into the REBA and other evaluation methods; it needs to be converted to a specific limb angle. The position of a joint point is defined as $p_{i}=\left(x_{i},y_{i}\right)$. Limb $l_{i}$ is expressed as a vector composed of joint points for both ends of the limb; thus, $l_{i}=\vec{p_{i}p_{i+1}}$. The absolute limb angle $\theta_{i}$ of limb $l_{i}$ is:

(1) $$\theta_{i}=\tan^{-1}\frac{y_{i+1}-y_{i}}{x_{i+1}-x_{i}}$$

The absolute limb angle *i* calculated by HSR is based on the X-axis (horizontal) of the front end point of limbs. However, in the REBA method, the Y-axis (vertical) is used as the reference. Thus, it was necessary to rotate the coordinate system to obtain the X-axis (vertical). The absolute limb angle of $l_{i}$ was further adjusted as: $\theta_{i}=90{}^{\circ}\pm \theta_{i}$. The angle above the neck was +, and the angle below the neck was −.

In the legs, for example, the absolute angle of the lower leg is affected by the upper leg, so it was necessary to calculate the postural angle of the lower leg relative to the upper leg. The relative angle $\theta_{i}^{’}$ between limb $l_{i}$ and limb $l_{i-1}$ is:

(2) $$\theta_{i}^{’}=\cos^{-1}\frac{\vec{p_{i}p_{i+1}}\cdot \vec{p_{i-1}p_{i}}}{\left|\vec{p_{i}p_{i+1}}\right|\left|\vec{p_{i-1}p_{i}}\right|}$$

Through these methods, the limb angle calculated by HSR can be entered into HRC for the calculation of REBA posture score.

**Figure 3:**
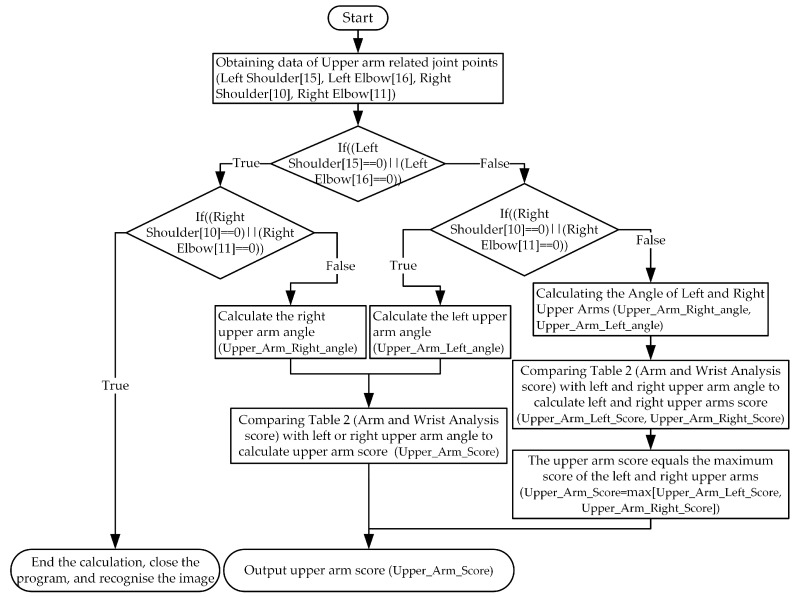
Flow chart of the algorithm for calculating the posture score of upper arm.

However, as the upper arm, lower arm, wrist, leg, and other parts of the body are present in both left and right groups, to accurately assess the risk of working posture, the system takes the maximum value of the two sides of the body posture score as the corresponding body assessment score. The algorithm flow of the upper arm is shown in Figure 3. After obtaining the data for joint points related to the upper arm, the system first determines whether the left and right joint points are recognized. If only one joint point is recognized, then the joint point is used to calculate the limb angle and the posture score. If both data are complete, then the left and right upper arm posture angles are calculated. The maximum value of the left and right upper arm posture angle was the upper arm posture angle score:

(3) Upper_Arm_Score=max[Upper_Arm_Left_Score, Upper_Arm_Right_Score]

where the Upper_Arm_Left_Score is the left upper arm angle score and the Upper_Arm_Right_Score is the right upper arm angle score. The formula can be used to score bilateral posture angles such as wrist, upper arm, lower arm, and leg in REBA, RULA, and National Institute of Occupational Safety and Health (NIOSH).

**Figure 4:**
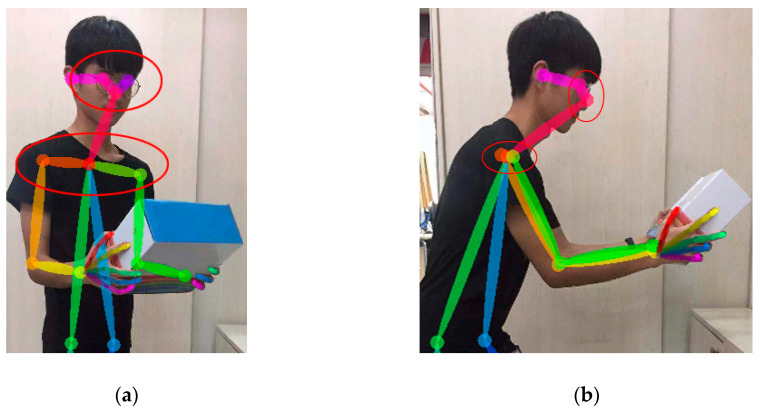
Compared with the exposed area of the chest and the face in the reverse state, (**a**) shows the waist and the head twisted state; (**b**) shows the normal non-twisted state.

The system assessment needs to consider the trunk and neck twisting state. A number of studies validated the difference between frontal and lateral exposure [4,41,42]. Based on the transverse distance of skeleton chest and face exposure, when the exposure ratio reaches 30%, the system can determine trunk or neck twisting as twisting and bending state (see Figure 4).

For the case of leg support, a variety of posture assessment have been studied [43]. When the difference between the two leg angles reaches 30 degrees, the system considers the leg position as a single leg support or kneeling, when the kneeling leg angle is greater than 90 degrees.

**Figure 5:**
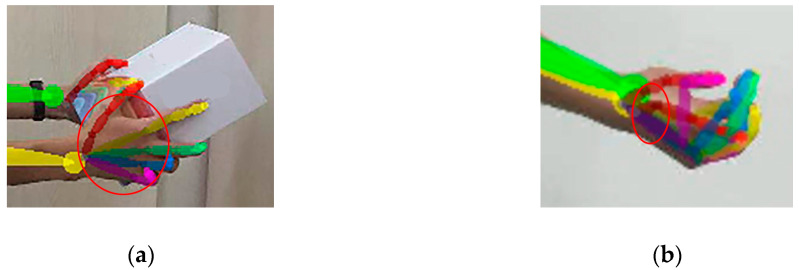
The normal status of the wrist (**a**) and the wrist flip status (**b**).

To evaluate the gravity-assisted scoring of the upper arm (upper arm score −1), the system first determines the bending of the trunk and upper arm. When the trunk bending is large and the upper arm bending is small, it can be considered that the upper arm score meets the gravity-assisted scoring [16]. To evaluate the problem of shoulder elevation (upper arm score +1), the system calculates the angle between the two lines connected by the neck, i.e., right and left shoulders. For wrist and arm twist problems, the system analyzes the hand exposure. As shown in Figure 5, when the exposure is small, the system considers that both wrist and arm are in the state of twist. When the arm is in normal condition, the system uses a twist angle instead of a flexion angle [16,32].

**Figure 6:**
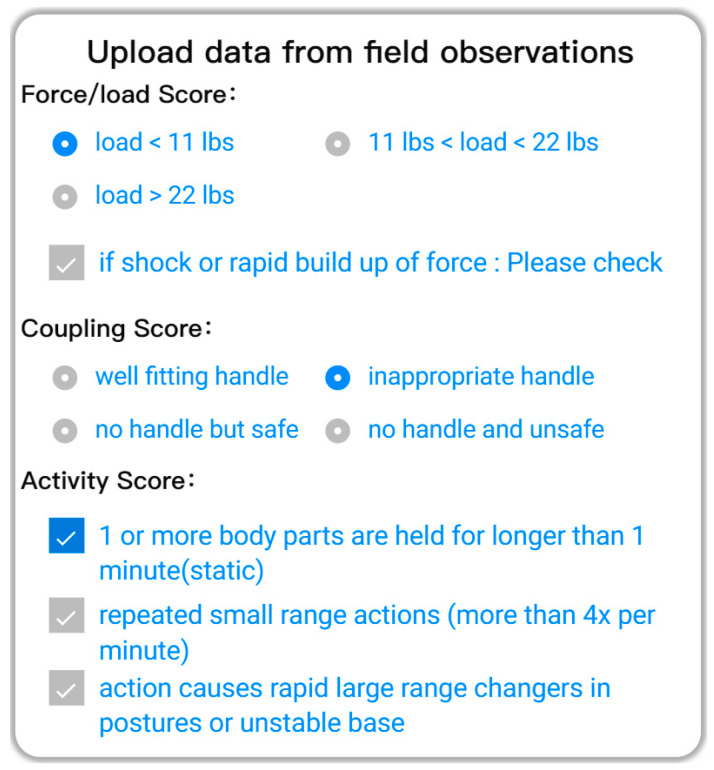
A screenshot of the interface for recording necessary data.

It is difficult for Quick Capture to assess other factors affecting REBA scores, such as force/load, coupling, and frequency, at the present stage. Thus, to solve these problems, simple options were set on the mobile side, as shown in Figure 6. As these three variables are recorded as operational observations, they are also inputted into the risk assessment system as known variables. The force/load score refers to task-related loads (0–3); coupling score describe the degree of ergonomic compliance of tool handles (0–3); frequency describes the operating frequency (0–3) [25].

### 2.2. Evaluating Experiment

The experiment was conducted in a laboratory at the South China University of Technology in October 2019. In this experiment, the reliability of the system was verified by studying the recognition capability agreement between the Quick Capture system and the motion capture system and the agreement between the Quick Capture system and the ergonomics expert, who verified the system’s feasibility. Thus, the experimental assumptions were as follows:

**Hypothesis** **H1.** *The limb angles recognition data of Quick Capture are consistent with the motion capture system.*

**Hypothesis** **H2.** *The evaluating scores of Quick Capture are in agreement with those obtained by the experts.*

#### 2.2.1. Participants

**Figure 7:**
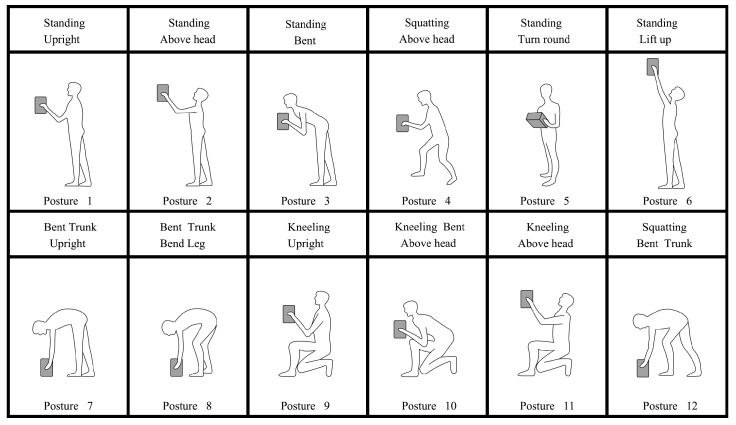
The 12 working posture using in the study.

A volunteer (male, 24 years old, 170 cm tall, and weighing 65 kg) was recruited to simulate the postures mentioned below (see Figure 7). The volunteer did not have any musculoskeletal and other physiological diseases and can independently complete tasks, such as handling and manual operations. In addition, three ergonomics experts with approximately ten years of human factor research experience related to MSDs were recruited. These experts have used the REBA evaluation method in research and teaching. Written consent was provided to the participants before the experiment.

#### 2.2.2. Equipment and Apparatus

To run the Quick Capture background server, it was connected to a notebook with a CPU Intel(R) Core (TM) i7-8750H 2.00 GHz, 8 GB RAM, GPU Nvidia GeForce GTX 1050Ti, and OS Windows 10, Made by Microsoft Corporation in Redmond, USA. The Quick Capture app was installed on a smartphone (iPhone XR Made by Apple Inc. in Cupertino, USA). The motion capture system was produced and installed by the Optitrack company in 2016. It is composed of six infrared digital cameras (Flex 13, with resolution up to 1.3 million pixels and number of frames of 120 FPS), a set of sports capture suits, a number of white marking points, and one PC (with a CPU Intel(R) Core(TM)i7-4790 3.60 GHz, 16 GB RAM, GPU Nvidia GeForce GTX 1050, and Windows 10 to run skeleton analysis software (Motive: Body 1.10.0 Final (64-bit) made by Natural Point, Inc. in Eugene, USA) and mark point capture accuracy reaching ‘submillimeter’).

A square carton (25 × 20 × 13 cm) was used to assist the volunteer in performing posture simulation and simulating the occlusion of the body using the tool employed in actual work. The simulation weight was 5 kg to prevent possible damage to the volunteer during the experiment.

Three laptops installed with ImageJ, Adobe Photoshop 2018 software, and the REBA forms were provided to the three experts so they could evaluate each working posture independently.

#### 2.2.3. Experimental Setting

Twelve static postures were selected for this experiment (see Figure 7) from the ergonomic assessment worksheet (EAWS) and the European campaign against musculoskeletal disorders [32,44]. These postures are common in workplaces.

**Figure 8:**
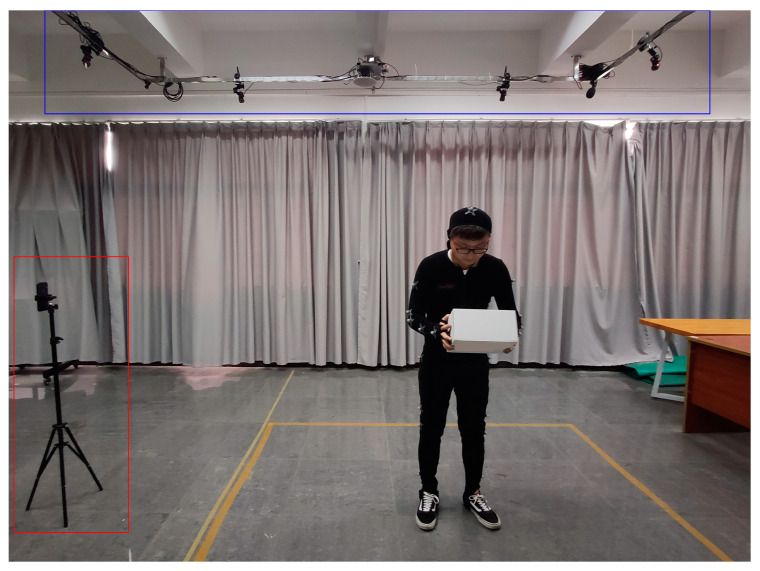
The experimental setting of this study.

The experimental environment is shown in Figure 8. The bottom calibration area was the recording area, which was at the center of the two systems to accurately capture positions. The volunteer was asked to wear an all-black sports capture suit. Twenty-one markers (1.0 cm diameter reflective spheres) were positioned on the anatomical point of the volunteer. For specifying landmark identification, Manghisi et al. [32] was used as the reference. After all the markers were completely attached, calibration of the motion capture system was performed before data collection. The Quick Capture system was positioned 2 m away from the sagittal plane of the volunteer and 1.3 m above the ground. A tripod was used to ensure the angles and heights at which the photos were taken by the smartphone were consistent. The top calibration area was the camera of the motion capture system. The experts were allowed to walk freely outside the yellow area to collect information on the working condition.

#### 2.2.4. Procedure

In the experiment, the volunteer was requested to perform one of the 12 postures to simulate the tasks randomly. Each working posture was maintained for 15 s, to ensure all the experts had observed and the value of the motion capture system was collected. The smartphone and motion capture system recorded the working postures simultaneously. After demonstrating the 12 postures, a 10-min rest was given to the volunteer. Then, the simulation was repeated following the above procedure. Overall, the simulation was repeated four times. After simulations were completed, the data recorded by the motion capture system for each posture were saved as a .tak file. In addition, the Quick Capture system automatically performed posture analysis and generated a risk assessment report for each posture. Moreover, the experts used computers to measure and calculate REBA risk levels for each posture independently.

#### 2.2.5. Data Analysis

All statistical analysis was performed using SPSS version 25.0 (IBM Corporation, Armonk, NY, USA). Prior to analyzing the data, a summary was created as follows: (1) extract the limb angle data for each posture from the .tak file saved in the motion capture system; (2) extract the limb angle data and REBA score for each posture from the assessment report collected by the rapid capture system; (3) summarize the REBA scores obtained by three experts.

To verify hypothesis 1, based on the coordinate data of the motion capture system, the ten limb angles, which were required by the REBA evaluation method, were calculated. A t-test analysis was applied to evaluate the difference between motion capture system and Quick Capture system under different limb parts. Finally, RMSE was used to compare the ten limb angles for each posture obtained by the Quick Capture system and the motion capture system. Subsequently, Spearman’s rho (ρ) correlation coefficient was used to compare the correlation of the two systems for each posture.

To verify hypothesis 2, the data from the three experts were used to calculate the intra-class correlation coefficient (ICC) for evaluating the reliability coefficient among observers [45]. The Quick Capture’s REBA score results were also used to calculate ICC. The closer the ICC value is to 1, the more reliable it is. The consistency between the expert and the system scores was compared by calculating the proportion agreement index (P_0_) and the strength of agreement on a sample-to-sample basis as expressed through linear weighted Cohen’s kappa [46,47].

## 3. Results

**Figure 9:**
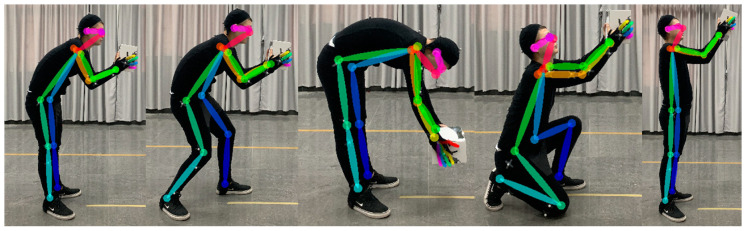
Human skeleton generated by Quick Capture system.

Figure 9 shows the part of the human skeleton generated by the Quick Capture system in the experiment. Human skeleton recognition was complete without pronounced misalignment. The evaluation data from the two systems and three experts were counted and used as a follow-up analysis. The original data of limb angles and REBA scores extracted are shown in Tables S1–S3 of the Supplementary Materials.

Table 2 reports the t-test results of the motion capture and Quick Capture systems. The results show that there was no significant difference between the two systems in recognizing each limb angle. To a certain extent, the Quick Capture can replace the motion capture system as a tool for limb angle recognition. In the limb angle of left lower arm, Quick Capture (50.595 ± 25.277) was different from the motion capture system (45.398 ± 26.327), but the difference was not significant; thus, further angle comparative analysis was necessary.

Table 3 reports the RMSE and ρ of all limb angles calculated through the Quick Capture and motion capture systems. These results show that the RMSE of the different calculated limb angles were very small. The average of RMSE from the 12 postures was 4.77, which ranged from 0.95 to 15.66. Posture 1 was the best case, which had an average value of 3.09 (with 9 angles among 10 and an error of less than 6). Posture 12 was the worst case, which had an average of 6.56 (with 4 angles among 10 and an error of less than 6). The correlation between Quick Capture and motion capture systems on limb angles was also analyzed. The average of ρ from the 12 postures was 0.915. In all selected postures, the correlations ranged from 0.726 to 0.988 and eight of the 12 postures were above 0.9. The results supported hypothesis 1, which stated that the limb angles data recognized by Quick Capture are consistent with those of the motion capture system.

The angles were also compared using the average of RMSE. Larger error was observed in the arm angle, especially the left lower arm (average 8.6). This could be due to the characteristics of elbows and wrists. They have a wide range of motion, are far from the torso of the body, and have relatively high instability. Thus, the cumulative and fluctuation errors may be large.

Table 4 reports the ICCs for the REBA scores. These scores were divided into REBA grand score, score A, and score B. The ICC results for the experts were very close to 1, which means that the three experts’ ratings were consistent. In addition, all the scores for the Quick Capture system’s ratings were very consistent (all ICCs > 0.973). Thus, the average of the three experts’ scores and the average scores of the system were further calculated to be used as the benchmark for subsequent evaluation.

**Figure 10:**
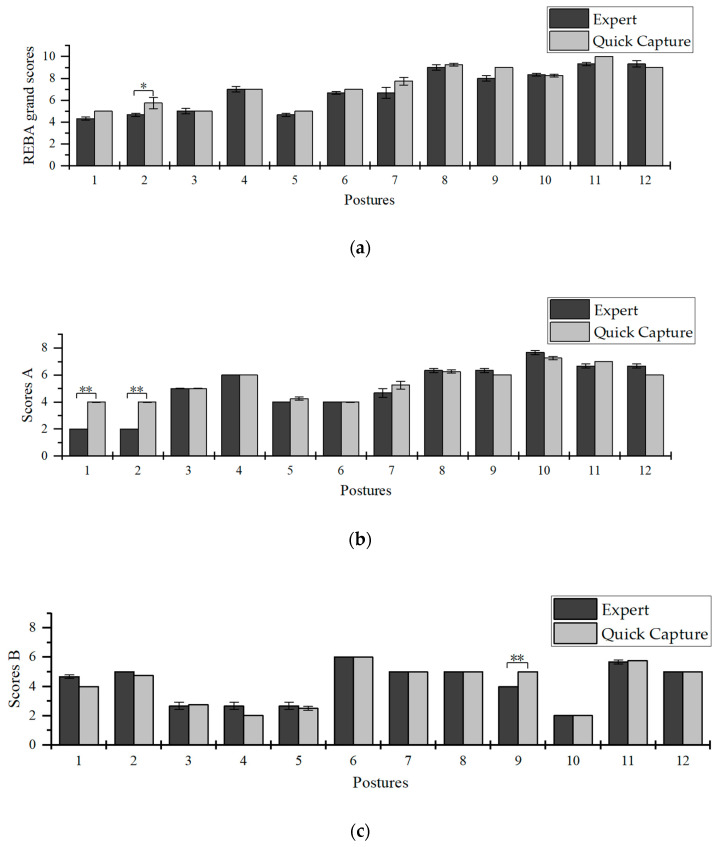
Comparison of REBA scores between experts and Quick Capture system evaluation. REBA grand scores (**a**), scores A (**b**), scores B (**c**). **: significant difference (*p* < 0.01); *: significant difference (*p* < 0.05).

Figure 10 shows the REBA scores for the Quick Capture in comparison with the average values for the three experts. The REBA grand score had a significant difference (*p* < 0.05) in posture 2. Score A had significant difference (*p* < 0.01) in postures 2 and 1. Score B had significant difference (*p* < 0.01) in posture 9. Overall, the REBA score obtained from this system was very close to the one obtained by the experts. The Quick Capture system can achieve expert-level scoring.

Table 5 reports the RMSE, P_0_, Cohen’s kappa, and the strength of agreement from the Landis and Koch scales [48] between the REBA scores computed using Quick Capture and the experts’ evaluation. For each REBA score, P_0_ was higher than 0.90 and Cohen’s kappa was higher than 0.7. These results indicate that there was a “substantial” agreement between the experts and the Quick Capture (*p* < 0.01, 0.6 < kappa < 0.8). Thus, the results indicate that the MSDs assessment by Quick Capture system was consistent with that by the ergonomics experts, and confirm hypothesis 2.

## 4. Discussion

### 4.1. Theoretical Contributions and Empirical Implications

The reliability and feasibility of the Quick Capture system was verified to prove its effectiveness. The study carried out a systematic laboratory evaluation, which quantitatively evaluated the accuracy of the Quick Capture system and motion capture system in recognizing limb angle and the consistency of the Quick Capture system with three experts on REBA scores. To the best of the authors’ knowledge, this is a new finding; thus, they proposed a theoretical model for enhancing accuracy of body postures. The main theoretical contributions of this research are as follows:

(1) The study applied a novel CPM-based REBA system for MSDs risk assessment, named the “Quick Capture“ system. To the best of the authors’ knowledge, this is the first system developed based on CPM-based REBA for MSDs risk assessment. This illustrates in-depth applications of CPM theory and the REBA system, and also enriches the adoption of the theory in image recognition in the field of ergonomics.
(2) To experimentally compare MSDs risk assessments, ergonomic experiments involving Quick Capture, ergonomic experts, and motion capture were conducted. The experimental design based on the Quick Capture system and the results of this study could provide considerable insights on MSDs risk assessment in the field of ergonomics.

Empirical implications are mainly in threefold:

(1) Quick Capture can demonstrate an automated mode on parameter-adjusting in REBA MSDs risk assessment. The scoring accuracy can also be improved.
(2) Quick Capture uses a smartphone as a carrier, which solves the tedious operations in the MSDs assessment. It also makes it possible to be a widespread application.
(3) The system can quickly complete MSDs assessments in real-life scenarios, thus, minimizing cost and time associated with MSDs assessment.

### 4.2. Summary of Expected Results

In the angle analysis, the average correlation of the postures was 0.915 (correlation ranged from 0.988 to 0.731), which was slightly higher than those obtained by Plantard et al. [47] (correlation ranged from 0.98 to 0.68). For the scores, 83.33% of the scores were consistent with those of the experts. P_0_ ranged from 0.968 to 0.931 and kappa ranged from 0.763 to 0.710, which were higher than those obtained by Abobakr et al. [39] (with an average P_0_ of 0.846 and ranged from 0.86 to 0.82). The average value of kappa was 0.700 and ranged from 0.78 to 0.63, while Plantard et al. [47] obtained P_0_ values ranging from 0.77 to 0.62 with an average of 0.712 and kappa values ranging from 0.66 to 0.46, with an average of 0.624. These results show that the Quick Capture system is not only robust in limb angle recognition, but also greatly improves the calculation of the REBA scores.

In addition, the RMSE of the left side of the body was found to be slightly higher than that of the right side. For example, the average values for the left leg, left upper arm, and left lower arm were 5.38 (3.26), 4.69 (3.11), and 8.6 (5.43), respectively. This could be due to the shooting from right to left during the experiment, which caused the right limb to occlude the left limb in the image and the weak left recognition effect. The side photo was selected for recognition, however, some occlusion errors still persisted. It is recommended to take pictures from the oblique side to eliminate occlusion errors. Further, it is possible to make an error comparison of multiple shooting angles and evaluate the occlusion error of the system at different angles.

Postures 3, 5, 6, 7, 8, 10, 11, and 12 were found to be in good agreement with the score data. Postures 1, 2, and 9 were slightly worse. Through the analysis of the original data, it was found that when the angle was close to the critical value, the noise and error generated during recognition increased. The findings were similar to those of Plantard et al. [47].

During data analysis, it was found that the differences between the system and expert evaluations were mainly reflected in the score A of postures 1 and 2 (Figure 10b). In posture 1 and posture 2, the score A given by the three experts were 2 points and the average score A from the system was 4 points. It was found that they were consistent in neck and leg scores, but there was a difference in the trunk threshold. The experts believed that the trunk angles in these postures are 0°, but the angles of neck and waist measured by the system were −0.89° (0.85°) and −10.2° (2.99°), respectively, which were not absolutely 0°. This was the cause in the difference for the neck and waist scores, resulting in the gap of score A. The Quick Capture system’s more objective image analysis method can make up for the possible errors made by experts.

In the total score, the system’s scores for the 10 postures were slightly higher than those of the experts. Quick Capture slightly overestimated the risk, but the overestimation was conservative [32]. Therefore, based on the characteristics of the system, it is suggested that when relevant experts use the system, they can judge whether the critical values have an essential impact based on the detailed reported data. For industry managers, the system can be directly used within the slightly higher risk estimation result. The risk assessment can provide useful information to workers for reducing work-related injuries.

Using Quick Capture will not directly interfere with the work of workers. It has no heavy supporting equipment. The evaluator has to use only the application installed in the smartphone for taking a complete picture of the worker’s work posture and enter some necessary data. After uploading, the phone will automatically send back a risk report based on the REBA rule, which considers more evaluation parameters and has higher accuracy. An evaluator can provide on-site improvement suggestions based on online reports or download detailed offline reports for in-depth analysis.

## 5. Conclusions

This study modified the automatic judgment of the trunk twist, leg support, wrist twist, etc., of the Quick Capture system. By using the motion capture system and utilizing experts as benchmarks, the reliability and feasibility of Quick Capture were verified. The results were compared to that of Abobakr et al. [39] and Plantard et al. [47]. It was proven that the Quick Capture system was slightly better than their results in terms of angle recognition, and its scores were higher than theirs.

Overall, it was proven under experimental conditions that:

(1) Quick Capture’s angle recognition accuracy was consistent with that of the motion capture system;
(2) The score calculated by Quick Capture was consistent with those of the experts;
(3) The Quick Capture system could make up for possible errors made by experts.

The results show that the system can be used as a rapid, automatic, and low-cost REBA analysis tool, without complex installation, deployment, and specific professional skills, and that the assessment process cannot interrupt the normal work of workers. Some limitations of the study and future research directions are discussed below. The study evaluated the Quick Capture system in a laboratory environment. The lighting conditions were good in the experimental environment and the occlusion of the tool was simulated. The capture suit worn by the volunteer in the experiment was tight and completely black, which had a particular simulation effect on working clothes. The effect of working clothes should be further investigated, especially loose and winter clothes. In addition, this study recruited a man to complete 12 postures and repeat it four times. A wider evaluation on different characteristics of participant will be necessary to increase representation for the entire workers, such as gender and body shape.

Currently, the system only evaluates the REBA method. However, in actual work, each evaluation method has its advantages and limitations [18]. Therefore, it is worthwhile to expand the evaluation method based on Quick Capture, analyze the similarities and differences of the multiple evaluation methods such as RULA, OWAS, and NIOSH in the evaluation process, and establish a multi-method comprehensive evaluation to meet the evaluation requirements of different labor sites. Other aspects, such as (1) further research works about REBA, MSDs, and occupational health research under the context of IoT-based industry transformation could be explored in-depth [9,48,49]. (2) How did IoT-driven Industry 4.0 environment affect workers’ operation with different kinds of product design and product fulfillment with the new manufacturing transformation of mass customization for high-complexity and low-volume production [50,51]? (3) When make-to-order (MTO) strategy makes future order fulfillment process more knowledge-centric and customized [51,52], will the job enrichment be positive for reducing the possibility of MSDs to create a better occupational health environment [51,52]? (4) More in-context ergonomic REBA and posture analysis with Artificial Intelligent-IoT (AIoT) can be further studied in-depth [50,53].

## Figures and Tables

**Table 1:** Rapid entire body assessment (REBA) grand scores, risk levels, and grade standard.

REBA Score	Risk Level	Risk Description
1	1	Negligible risk
2~3	2	Low risk. Change may be needed
4~7	3	Medium risk. Further investigate change soon
8~10	4	High risk. Investigate and implement change
11+	5	Very high risk. Implement change

**Table 2:** Results of t-tests between the motion capture system and the Quick Capture.

Body Parts	Mean (SD)		Significance
	Motion Capture System	Quick Capture System	
Neck	−6.178 (12.455)	−6.072 (10.790)	NS
Trunk	36.066 (34.010)	32.793 (32.628)	NS
L-Legs	39.802 (48.076)	42.796 (48.690)	NS
R-Legs	52.839 (59.726)	51.314 (60.963)	NS
LU-Arm	43.974 (39.558)	43.204 (40.474)	NS
RU-Arm	39.413 (39.322)	41.975 (39.657)	NS
LL-Arm	45.398 (26.327)	50.595 (25.277)	NS
RL-Arm	53.156 (25.459)	55.236 (26.058)	NS
L-Wrist	6.983 (4.634)	6.404 (4.435)	NS
R-Wrist	8.462 (5.272)	7.888 (4.201)	NS

NS—no significant difference; L—left; R—right; LU—left upper; RU—right upper; LL—left lower; RL—right lower; SD—standard deviation.

**Table 3:** Comparison of RMSE (+SD) and ρ, and consistency between Quick Capture and the motion capture system.

RMSEs	Body Parts										AVE	ρ
	Neck	Trunk	L-Legs	R-Legs	LU-Arm	RU-Arm	LL-Arm	RL-Arm	L-Wrist	R-Wrist		
1	0.95(0.98)	2.28(2.42)	2.83(1.61)	2.34(1.38)	5.50(3.13)	4.29(3.14)	4.83(3.32)	3.36(3.06)	2.73(1.90)	1.71(1.88)	3.09	0.896 **
2	4.89(5.62)	3.12(2.05)	3.20(1.00)	2.25(2.60)	1.98(0.66)	2.13(0.26)	5.17(3.03)	5.27(5.76)	3.08(1.84)	5.53(3.11)	3.66	0.968 **
3	1.86(0.84)	3.03(2.29)	2.66(2.00)	4.04(0.91)	3.12(2.64)	1.91(1.89)	9.70(7.98)	5.10(5.50)	3.10(2.53)	3.50(2.78)	3.80	0.963 **
4	3.33(2.36)	4.27(1.30)	1.49(1.60)	6.49(3.11)	2.64(2.99)	3.10(3.19)	5.06(4.37)	4.45(4.43)	3.57(2.47)	5.09(2.92)	3.95	0.988 **
5	3.72(3.23)	3.29(0.52)	2.63(2.16)	2.02(1.71)	5.14(4.25)	3.70(1.54)	7.58(1.26)	5.34(1.94)	4.14(3.02)	4.79(3.61)	4.24	0.824 **
6	4.24(3.09)	2.03(2.07)	1.73(1.15)	3.38(0.74)	4.38(3.50)	5.58(3.76)	10.76(6.3)	7.55(4.11)	2.86(3.16)	2.37(1.86)	4.49	0.963 **
7	3.73(3.58)	7.90(0.93)	7.63(4.73)	4.26(4.73)	5.59(1.43)	4.51(4.61)	8.21(8.01)	4.42(4.95)	3.39(3.90)	4.64(1.48)	5.54	0.726 **
8	4.96(4.96)	4.43(2.02)	9.06(6.25)	2.87(1.88)	3.15(3.19)	5.10(1.17)	15.66(8.65)	4.24(0.03)	2.18(2.42)	4.96(2.72)	5.78	0.874 **
9	4.44(4.77)	4.10(2.29)	7.26(5.60)	1.98(1.83)	4.40(4.10)	4.81(4.28)	5.11(5.74)	5.01(4.89)	2.72(2.27)	4.59(1.50)	4.56	0.980 **
10	3.11(3.29)	1.94(1.44)	8.55(6.92)	3.63(1.49)	11.68(2.09)	5.62(1.46)	12.81(9.96)	5.05(4.9)	4.29(4.95)	4.41(4.73)	6.25	0.959 **
11	2.38(2.70)	3.53(0.77)	10.95(3.33)	3.46(3.94)	3.43(3.39)	4.55(2.33)	11.07(2.76)	5.12(3.22)	3.70(4.22)	4.94(5.38)	5.31	0.983 **
12	5.41(2.07)	6.40(5.04)	6.55(2.78)	4.53(1.34)	5.26(6.01)	10.3(1.45)	7.20(3.80)	6.82(3.26)	7.31(6.92)	5.55(5.00)	6.56	0.850 **
**AVE**	3.58	3.86	5.38	3.44	4.69	4.63	8.60	5.14	3.59	4.76	4.77	0.915

L—left; R—right; LU—left upper; RU—right upper; LL—left lower; RL—right lower; AVE—average; **—significant difference (*p* < 0.01). RMSE—root mean squared error. The numbers 1–12 on the left in Table 3 represent the serial numbers of the 12 working postures.

**Table 4:** ICC results of experts and Quick Capture.

ICCs	Quick Capture	Expert
REBA Grand Score	0.980	0.961
Score A	0.973	0.981
Score B	0.989	0.926

**Table 5:** Comparing linear weighted Cohen’s kappa, P_0_, and RMSE, consistency between Quick Capture and expert evaluation was observed.

	RMSE	P_0_	Cohen’s Kappa	*p* Value
REBA Grand Score	0.622	0.968	0.710	<0.01
Score A	0.878	0.931	0.742	<0.01
Score B	0.408	0.957	0.763	<0.01

## Supplementary Materials

The following are available online at https://www.mdpi.com/1424-8220/20/16/4414/s1. Table S1: The motion capture system; Table S2: Quick Capture system; Table S3: REBA grand score.

## Appendix B

The following is the link to the code of this article:

https://github.com/xiaozeguo123/-Quick-Capture-system-HSR.
